# The practical utility of genetic screening in school settings

**DOI:** 10.1038/s41539-021-00090-y

**Published:** 2021-06-01

**Authors:** J. Shero, W. van Dijk, A. Edwards, C. Schatschneider, E. J. Solari, S. A. Hart

**Affiliations:** 1 Department of Psychology, Florida State University, Tallahassee, FL USA; 2 Florida Center for Reading Research, Florida State University, Tallahassee, FL USA; 3 Department of Curriculum Instruction and Education, University of Virginia, Charlottesville, VA USA

**Keywords:** Education, Interdisciplinary studies

## Abstract

Can genetic screening be used to personalize education for students? Genome-wide association studies (GWAS) screen an individual’s DNA for specific variations in their genome, and how said variations relate to specific traits. The variations can then be assigned a corresponding weight and summed to produce polygenic scores (PGS) for given traits. Though first developed for disease risk, PGS is now used to predict educational achievement. Using a novel simulation method, this paper examines if PGS could advance screening in schools, a goal of personalized education. Results show limited potential benefits for using PGS to personalize education for individual students. However, further analysis shows PGS can be effectively used alongside progress monitoring measures to screen for learning disability risk. Altogether, PGS is not useful in personalizing education for every child but has potential utility when used simultaneously with additional screening tools to help determine which children may struggle academically.

## Introduction

Websites such as 23andMe.com and Ancestry.com have gained increased popularity in recent years as individuals are getting more interested in what their DNA says about their ancestry and about some of their traits. Although these commercial ancestry genetic tests are what many people are most familiar with, the uses for similar genetic screening are rapidly expanding^1^. Though controversial for reasons related to demographic disparities, fear, and genetic essentialism^2,3^, research and use of genetic screening has become more established in the medical field, leading to what is known as “precision medicine”. Precision medicine aims to tailor prevention and treatment based on an individual’s genes, as well as environments, and lifestyles^4^.This framework of approaching the treatment and prevention of biomedical disease using genetic information has been used with some success^5^ for both monogenic disorders, as well as disorders with more complex polygenic patterns such as psychiatric disorders^6^. Assessing the genetics underlying these more complex traits is typically done using polygenic scores (PGS)^7^. These PGS are developed from genome-wide association studies (GWAS) that screen an individual’s DNA for specific variations in their genome, and the relations said variations have with the specific trait. Said variations are then assigned a corresponding weight and summed to produce a PGS for a given trait. Under the precision medicine framework, PGS have been used for estimating the risk of acquiring certain conditions or diseases^8–11^. More recently, similar PGS methods have been used for predicting social outcomes^12^, such as educational attainment^13^, personality^14^, and socioeconomic status^15^. This extension of genetics into social sciences, including into educational science, has garnered excitement and controversy, with some saying that we can mirror the precision medicine movement in education to “personalize education” by personalizing instruction and prevention based in part on genes^16,17^. Though there are many definitions for what personalized education means (differentiated at the individual or group-level, self-paced or self-guided, using educational technology, etc.), here we define personalized education as differentiated and targeted instruction at the individual level. Educationally relevant outcomes have many parallels to biomedical outcomes, including individual differences characterized by quantitative patterns of genetic, environmental, and psychological risk and protective factors, making the extension of precision medicine into education seemingly a useful next step. There are also important differences, including biomedical research having a better grasp of mechanisms of disease, more clear definitions of disease, and fewer concerns of determinism and genetic essentialism—the idea that genes are deterministic. Importantly, as part of the personalized education framework, educators would use various tools and screeners, which could include genetic information via a PGS, to better understand individual students, and provide a personalized education experience that targets each student’s needs. Said approach would have to keep in mind that PGS results are only one (currently small) aspect that impacts development and are not deterministic, but rather that the information gained can be used alongside other information to better predict general trends in educational achievement for individual students. With this in mind, the idea of personalized education is thought-provoking and comes with its own share of criticisms, including considerable ethical concerns regarding genetic essentialism and inequitable effectiveness, as well as a lack of portability of results across races and ancestry groups making current genetic technology limited in utility outside of European ancestry groups^18^. These criticisms withstanding, the practical usefulness of using genetic information as part of a personalized education framework has not actually been tested. It is not clear if genetic technological capabilities support the idea that using PGS would add incremental value to the universal screening system for educational risk already in place in many schools, or do so to the extent the outweighs ethical concerns. In this paper we quantify the incremental value of using PGS in tandem with behavioral screening measures for academic achievement and risk, moving beyond the conceptual idea of a genetically informed personalized education, to explore two research questions. First, what incremental value could a PGS have in predicting student academic achievement? That is, determining if PGS could be used as part of a battery of tools to differentiate students based on their DNA. Specifically, seeing if schools would be able to use PGS to increase their ability to predict the end of the year achievement beyond currently used progress monitoring tools, and therefore, help them better differentiate instruction to target student needs. Second, could PGS be used as part of a screener to increase accuracy in identifying students at risk of developing a learning disability? In other words, testing if PGS could be used as part of a screener to better identify children who might struggle. We purposely do not limit our analysis to a specific kind of academic achievement or learning disability (e.g., reading, dyslexia), or to general achievement (e.g., grade point average), but instead keep our modeling flexible enough to represent the range of possible uses for PGS in schools. This allows us to more broadly consider how PGS might be used as part of personalized education across multiple academic content areas. Further, it should be noted that the results to these questions are not deterministic, but rather aim to identify PGS’s ability to predict more potential trajectories in educational achievement rather than predicting a predetermined or fixed level of achievement.

Accurately identifying current student performance and using that to predict their expected later outcomes is essential to meet the instructional needs of individual students. In attempts to accurately predict student achievement, and risk for developing learning disabilities, many schools have implemented various systems to universally screen students. At the core of comprehensive universal screening, systems lie progress monitoring or frequent evaluation of students to determine which students need supplemental intervention^19^. The primary method used for screening is evaluating student performance once to several times a year using progress monitoring tools (monthly, every other month, fall/winter/spring, etc.). This includes the more novel progress monitoring tools such as computer adaptive testing that have recently become more popular^20^. Screening using progress monitoring tools in this way is used to determine if at-risk children need supplemental intervention and to predict how a child might perform on end-of-the-year standardized achievement tests. As such, the purpose of screening and progress monitoring tools is to provide teachers the necessary information to guide targeted instruction to students. Specifically, when considering students who are at risk for difficulties, it is important to accurately identify them at the beginning of the school year so that adequate differentiated instruction can be provided. Unfortunately, many of the commonly used progress monitoring tools have moderate predictive validity leading to low classification accuracy, often resulting in 25–60% misclassified students^21,22^. Current estimates show that these progress monitoring tools correlate with end of the year scores at a range of about 0.40–0.70, and as such, we used values representing this range (*r* = 0.40, 0.55, 0.70) as estimates of effectiveness for our analyses. This low classification accuracy means either some students will receive intervention unnecessarily, depleting school resources, or some students will not receive the supplemental intervention they need and fall further behind. Classification accuracy can be increased by combining multiple progress monitoring tools, or follow-up intensive assessments^23,24^, methods that potentially expend school resources. As such, the incremental value of PGS will be examined as an additional tool for universal screening systems that could be used to increase prediction accuracy. To address the research questions presented above, it is important to assess PGS’s potential incremental value alongside progress monitoring tools. Only then, will we be able to see what new value it adds, and determine where it could fit as a tool for educators in authentic school settings.

Recent studies on PGS in educational settings have shown that currently, PGS can predict a composite academic achievement measure, educational attainment, and reading comprehension abilities with up to 10%, 15%, and 5% accuracy, respectively^13,25–27^. As molecular genetics methods get better and reference sample sizes get bigger, the predictive power of PGS to educationally relevant outcomes are expected to substantially increase, with some research estimating predictive limits as high as about 50% in student outcomes^13,28^. However, adding to the complexity of testing the incremental value of using PGS as part of personalized education, the current and hypothesized future predictive values of PGS are currently debated. It has been argued that the potential upper limit of PGS predictive effectiveness is much lower than 50% as twin estimated heritability is thought to be overinflated due to violations regarding the assumptions of equal environments^29^. In addition, differences in the predictive effectiveness of PGS are dictated by the characteristics of the individuals used to create the reference datasets to calculate the PGS^30^, and the populations they are being used to predict. For example, to date, around 79% of the reference samples used to create a PGS are drawn from European ancestry populations^31^. When using a PGS drawn from these majority European ancestry populations to predict outcomes in individuals who do not come from those same ancestry groups, like ~45% of the student population in the US^32^, the accuracy of prediction is greatly diminished^31^. This is due to genetic variants being more common among some ancestry groups than others, and as such the significant variants found in one population not translating as well for outside populations. Adding to the concerns of less predictive power from PGS for individuals from non-European ancestries, children from racial minority backgrounds in the US are already less likely to be identified by current screening systems for special education services (e.g., having a learning disability)^33^. This would mean that adding PGS, as the current technology stands, into personalized education would likely further exacerbate educational disparities. Due to the vast majority of literature being conducted using European ancestry groups, the results of this paper should be interpreted with students of European ancestry in mind. For students of differing ancestry groups the results of this study are likely to be far less due to the lower effectiveness of PGS across ancestry groups^18,33^, and again points to the need for more research on populations of non-European ancestry^34^. In addition, PGS are probabilistic, not deterministic, and there is reason to think that the environments students are in would influence the role their genetic variants have on their educational outcomes, although the work in this area is just starting^35^. One such theory on how this could work is an individual’s genetic variants can make that individual more or less susceptible to the environments around them, with genetic influences being negatively expressed in detrimental environments and beneficial expressed in supportive environments^36^. The predictive effectiveness of PGS can also vary based on student age and academic measure of interest. For those studies focused on older students and measuring composite or more general measures of achievement, the current estimates for PGS effectiveness are as high as 15% of the variance in these academic measures explained^27^. However, studies that have focused on younger students and more specific measures of academic achievement (e.g., reading comprehension) had considerably lower estimates of effectiveness ranging around 2–5% of variance explained^25^.

As it stands now, previous research has examined the extent to which PGS can predict variance beyond that which is predicted by environmental factors such as socioeconomic status, but schools and districts rarely use this type of information in attempts to predict individual student performance. Rather, teachers and schools use a variety of progress monitoring tools as part of a universal screening system to make these predictions, but to this point, research has not examined what value PGS could add as an additional tool in the universal screening system. This paper aims to conduct said examination and provide insight into what PGS might look like alongside the progress monitoring tools regularly being used in schools^12,25,27,37,38^. To reiterate our two research questions from above, first, what incremental value could PGS have in predicting student achievement? And second, could PGS be used as a tool to increase accuracy in identifying students at risk of developing a learning disability? The methods taken to answer the two research questions are as follows. For the first research question, a combination of data-simulation and regression techniques were used, simulating data and determining the increased level of variance in end-of-the-year achievement scores (e.g., standardized tests) predicted when adding in PGS alongside existing progress monitoring tools. The second question was explored using probability density functions to determine how much overlap there would be between progress monitoring scores, PGS, and end-of-the-year achievement scores at various common cutoffs for learning disability classification (5th, 10th, 15th, and 20th percentiles). For both questions, data were simulated and density functions computed using 12 combinations of progress monitoring scores’ correlations to end of year scores (*r* = 0.40, 0.55, 0.70), and PGS’s ability to predict variance at the end of year scores (2%, 10%, 20%, and 50%). These values for PGS effectiveness were chosen from a combination of educational achievement measures representing a realistic range of different student achievement values, including the 2% value representing early grade reading comprehension^25^, 10% and 20% values representing a range of current estimates of general educational achievement and intelligence and an estimated limit of where PGS for more specific measures will get in the near future^13,25,27^ and 50% representing an estimated theoretical threshold from twin studies^25^. As mentioned prior, however, each of these values is up for debate, and we do not wish to speak to only one narrow definition of student achievement, and as such results for PGS effectiveness in a wider range (2%, 10%, 20%, 30%, 40%, 50%, 60%, 70%, and 80%) were tested as a supplement for one of the models related to each research questions. If PGS were to ever predict more than 80% of achievement, they would no doubt be useful if ethical concerns were adequately addressed. Further, for correlational values of progress monitoring to student outcomes, predictive values of PGS to student outcome, and potential cutoffs to characterize learning disabilities, not represented in this paper, a ShinyApp (https://jshero.shinyapps.io/two_predictor_screener/) was developed allowing any combination of correlations among the variables to be tested as a screener for any cutoff in the same ways presented in this paper.

## Results

### Research question 1

Table 1 shows the overall amount of variance predicted in end-of-year achievement by progress monitoring scores and PGS, dependent on the given effectiveness of each, as well as the unique variance predicted by PGS. Results of these simulations indicate that when the predictive value of PGS is low (i.e., where the technology is currently, 2%), using the PGS adds very little in terms of the overall variance in achievement predicted, even when progress monitoring’s predictive value is also low. However, as the predictive value of the PGS gets higher, so too does its overall contribution to variance predicted growth. At 10% and 20% predictive power, PGS predicts as much as 4% and 8.9% additional variance in achievement respectively when progress monitoring is at its least effective, and as much as 1–2.3% respectively additional variance for the highest estimates of progress monitoring effectiveness. At the theoretical limit of 50% predictive power, additional variance predicted by PGS reaches as much as 35.9% when the predictive value of progress monitoring is low, and 9% even when the predictive value of progress monitoring is high. Results of analysis for additional values are presented in Supplementary Table 17.

**Table 1:** Variance in end-of-year achievement predicted by progress monitoring and PGS.

	Variance at the end of year achievement predicted by progress monitoring and PGS		
	Progress monitoring effectiveness		
PGS Predictive Power	**0.40**	**0.55**	**0.70**
**2%**	0.167 (0.007)	0.3066 (0.004)	0.492 (0.002)
**10%**	0.199 (0.040)	0.325 (0.023)	0.500 (0.010)
**20%**	0.249 (0.089)	0.353 (0.051)	0.513 (0.023)
**50%**	0.5198 (0.359)	0.505 (0.203)	0.580 (0.090)

*R*^2^ values presented, with the unique contribution of *R*^2^ by PGS presented in parentheses.

### Research question 2

As stated prior, four separate cutoffs for learning disability were examined in this section of the paper (5th, 10th, 15th, and 20th percentiles). Since a diagnosis of a learning disability is not something that can be scientifically tested, finding students that fall in the extreme low ends of the distribution is one typical way to classify learning disabilities and was the definition of learning disabilities used here. Results from one cutoff (tenth percentile), are presented in Tables 2–4. Table 2 presents the current state of screening for learning disabilities, showing positive and negative predictive values (NPVs) for progress monitoring at three different levels of effectiveness. positive predictive values (PPVs) represent the percentage of students identified as being at-risk for having a learning disability that actually had a learning disability, and the NPV represents the percentage of students that were identified as not at-risk that did not develop a learning disability. For example, when progress monitoring is highly effective (*r* = 0.70) 34.5% of students who were identified as being at-risk were later identified as learning disabled, and 96.1% of students who were considered not at-risk did not develop a learning disability. These values serve as a baseline for comparison when analyzing the incremental value of using PGS. Results of analysis for additional values at the 20th percentile cutoff are presented in Supplementary Tables 18 and 19.

**Table 2:** Positive and negative predictive values of progress monitoring tools as a screener for learning disabilities (tenth percentile or lower achievement).

Predictive power of only progress monitoring		
Progress Monitoring		
**0.40**	**0.55**	**0.70**
21.9% (92.9%)	27.8% (94.4%)	34.5% (96.1%)

The positive predictive value presented as a percentage with negative predictive values presented as a percentage in parentheses.

**Table 3:** Positive and negative predictive values of progress monitoring tools and PGS (meeting either’s criteria) as a screener for learning disabilities (tenth percentile or lower achievement).

	Predictive power when meeting either cutoff		
	Progress monitoring		
PGS Predictive Power	**0.40**	**0.55**	**0.70**
**2%**	16.7% (93.6%)	19.1% (94.9%)	22.0% (96.4%)
**10%**	18.6% (94.2%)	20.9% (95.4%)	23.6% (96.8%)
**20%**	20.3% (94.9%)	22.5% (95.9%)	25.1% (97.0%)
**50%**	25.8% (96.3%)	27.2% (96.9%)	29.3% (97.8%)

The positive predictive value presented as a percentage with negative predictive values presented as a percentage in parentheses.

**Table 4:** Positive and negative predictive value of progress monitoring tools and PGS (meeting criteria of both) as a screener for learning disabilities (tenth percentile or lower achievement).

	Predictive power when meeting both cutoffs		
	Progress monitoring		
PGS predictive power	**0.40**	**0.55**	**0.70**
**2%**	26.0% (87.7%)	31.8% (88.1%)	38.4% (88.7%)
**10%**	30.2% (88.8%)	35.9% (89.4%)	42.4% (90.1%)
**20%**	32.7% (89.8%)	38.4% (90.5%)	44.8% (91.2%)
**50%**	35.1% (92.0%)	41.4% (92.9%)	47.9% (93.9%)

The positive predictive value presented as a percentage with negative predictive values presented as a percentage in parentheses.

Table 3 presents the positive and NPVs of these tools as a screener for when anyone who meets the cutoff for either progress monitoring or PGS is identified as being at-risk. Results from this model show that introducing PGS as a secondary method of being identified as at-risk lowered positive predictive while raising NPV for every situation except when PGS reached its theoretical limit and progress monitoring was at its least effective. This strategy would be effective for schools that prefer broader ranges of cut-offs so as to reduce the false-negative rate. Even in this case, the boost to PPV was only 3.9%. However, an increase in NPV was consistent in every situation. That is, the addition of PGS cutoffs as a way of being classified as at-risk led to fewer false negatives at all levels of PGS effectiveness. The higher the effectiveness of PGS the greater the increase to NPV and the greater the decrease to false negatives. The increase to NPV ranged from 3.4% when progress monitoring was least effective and PGS was at its theoretical limit, to 0.3% when progress monitoring is highest and PGS effectiveness is weakest. As PGS gets more accurate, the extent to which it lowers PPV is decreased while its impact on NPV increases. However, as the predictive value of progress monitoring gets higher, the incremental value of PGS decreases. Table 3 shows the results only at the tenth cutoff definition for learning disability, however, for all cutoffs these same patterns emerge (Supplementary Tables 3, 7, 11, and 15).

**Fig. 1:**
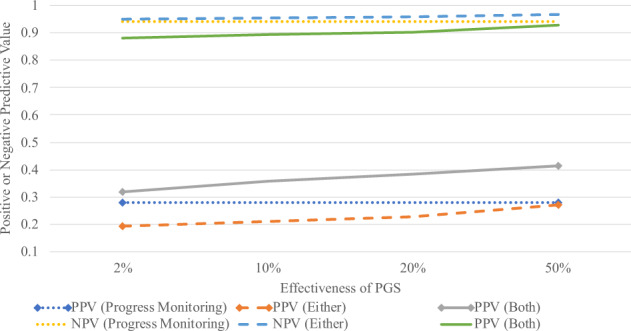
Trends in positive and negative predictive values of using PGS and progress monitoring as a screener for learning disabilities (tenth percentile cutoff) at varying levels of PGS effectiveness. This table represents when progress monitoring is correlated with end-of-year achievement at *r* = 0.55. PPV positive predictive value, NPV negative predictive value.

Table 4 presents the positive and NPVs of these tools as a screener for anyone who is identified as being at-risk by meeting cutoffs for both PM and PGS. Results demonstrate that at every combination of PGS and progress monitoring effectiveness the requirement to meet both PGS and progress monitoring cutoffs decreases the NPV and increases the PPV. This strategy would be effective for schools that prefer a stricter classification for being at-risk so as to reduce the false-positive rate. Again, the extent to which it does this varies largely on the effectiveness of each measure. Increases in PPV ranged from 3.9% to 13.4%, whereas decreases in NPV ranged from 0.9% to 8.2%. As the predictive value of PGS increases, its impact on PPV increases, and its lowering effect on NPV decreases. In relation to the predictive value of progress monitoring, when the predictive value of progress monitoring is the lowest the decrease in NPV is also lowest, and when the predictive value of progress monitoring is highest, so too is the negative impact of the addition of PGS has on NPV. Figure 1 shows these trends for both methods of utilizing PGS alongside progress monitoring as a screener, using the middle correlation of 0.55 and showing the differing positive and NPVs at each value of PGS effectiveness. For cutoffs or values of progress monitoring and PGS not represented in this paper, a ShinyApp (https://jshero.shinyapps.io/two_predictor_screener/) was developed allowing any combination of correlations among the variables to be tested as a screener for any cutoff in the same ways presented here.

**Fig. 2:**
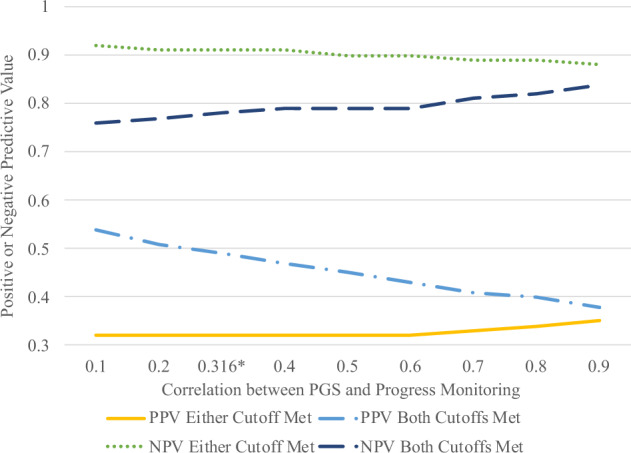
Positive and negative predictive values of using PGS and progress monitoring scores as a screener for learning disabilities (20th percentile cutoff) with varied correlations between PGS and progress monitoring. This table represents when PGS predicts 10% of end-of-year achievement scorers and progress monitoring is correlated with end-of-year achievement at *r* = 0.55. PPV positive predictive value; NPV negative predictive value. *Represents the value reported in the article where the relation between PGS and progress monitoring is equal to the relation between PGS and achievement.

One potential issue is the assumption that PGS correlates the same with progress monitoring as it does with end-of-the-year achievement. To show how contradictions to this assumption would impact the overall results, Fig. 2 was developed that shows positive and NPVs for one level of effectiveness of PGS (10%) and progress monitoring (*r* = 0.55), but with varying correlations between progress monitoring and PGS. This showed that a stronger correlation between progress monitoring and PGS leads to lower PPV and higher NPV when meeting both cutoffs. This is because, at higher correlations, these two variables are capturing more of the same variance in end-of-the-year achievement scores than when the correlation between the two is lower. When meeting either cutoff, changes in the correlation have far less impact and lead to slightly lower positive and NPVs as the correlation gets higher. Regardless of the changes seen here, however, the same trends still emerge with PPV increasing and NPV decreasing when making students meet both screener cutoffs, and vice versa when allowing students to meet either screener cutoff to qualify as at risk for a learning disability.

## Discussion

These results have powerful implications for personalized education, questioning if there is any incremental value for genetics in school settings. From the first research question, when considering predicting the overall end-of-year academic achievement, the addition of PGS *might* add some value. However, when PGS only predicts about 2% in variance in student outcomes (i.e., when defined as specific measures of academic achievement and for younger children, or a lower bound of effectiveness for non-European ancestry individuals), PGS adds very little in terms of predicting general variance at the end of year achievement beyond progress monitoring. This aligns with what was found by Morris and colleagues^39^, who found limited utility using PGS to predict achievement for individual students. However, as PGS reaches the 10% and 20% values that represent the current predictive power for broader measures of academic achievement and intelligence for older populations, and current PGS technology’s limit for predicting intelligence, the value PGS adds to predicting the end of year achievement becomes larger. At just 10% predictive power, PGS predicts an additional 1–4% variance in end-of-year achievement. Higher levels of PGS predictive power showed increases in this variance predicted across the board, and if PGS is ever able to reach the theoretical threshold of 50% tested in this paper, it could predict as much as an additional 36% at the end of the year achievement. How much given schools value these increases is entirely dependent on the values and views of those schools, but regardless there does appear to be some incremental value, when defined as variance predicted, to the future use of PGS in a school setting. The extent to which this is true additionally hinges on the type and goal of the PGS used, with more specific measures showing less potential benefit than general measures of achievement.

The results of the second research question showed there is some value in the addition of PGS as a screener of learning disabilities alongside progress monitoring. Results showed that adding PGS as a screener could be strategically used to increase either positive or NPV. The choice for how to use these scores would depend on the values and views of the school or locality. If a school’s primary aim was to decrease the number of students missed by current progress monitoring screeners, then allowing students to be identified as at-risk by either progress monitoring or PGS would do just that. This may be especially beneficial in earlier grades when early identification is key to ensuring optimal intervention efficacy for student growth^40,41^. On the other hand, if schools were more interested in saving resources and ensuring those students identified as at-risk and receiving additional services were actually students who would develop a learning disability, then labeling only students identified by both measures as at-risk would also serve to just this.

These results, combined with those from Morris^39^, indicate the best path to using PGS in school settings may not be through personalized education as we defined it, as allowing for differentiated instruction at the individual level. Personalized education requires us to be able to predict with a large degree of accuracy where a student will fall at the end-of-year achievement distribution, something the first research question showed PGS is not capable of doing at this point. Further, this suggests that with the limited understanding we have of how genetics impact learning and educational achievement, there is simply not enough evidence out there to support the idea of personalized education using genetics as many have suggested. However, using PGS in different ways, such as part of a screener for learning disabilities, would allow PGS to be used in a beneficial way without requiring the same high degree of predictive accuracy. Again, this assumes the definition of personalizing education as being at an individual student level. Personalizing education at a group level does require less precision and using PGS in this fashion could have some utility. In this case, rather than aiming to differentiate to each individual’s specific needs, predicting more broadly which quartile or quantile of achievement a student will fall in could help to differentiate based on group needs rather than individual needs, and is able to be done without requiring as much precision as individual differentiation. This is because grouping can occur with a wider margin of error, and approximations of where a student will fall are acceptable in contrast to differentiation at the individual level which requires more precision. However, the low levels of variance predicted by PGS and the fact that PGS on its own cannot be used to diagnose learning disabilities make it unlikely that on their own they will ever be able to do this, but in conjunction with other screeners as PGS get stronger they may become useful towards personalizing education at this broader group level.

Beyond understanding variance predicted and positive and negative predictive power, the question of time and monetary cost to school when adopting PGS is also important. The use of another progress monitoring tool, such as a different behavioral assessment, could lead to similar results as using a PGS. However, using additional progress monitoring tests could take a considerable amount of instructional time. Many progress monitoring tests take as long as 30 min to complete excluding the indirect time cost associated with organizing the classroom and situating students to complete the assessment^42^. Although some progress monitoring tools report immediate results, this is not the case for all. This could lead to additional time for grading assessments by teachers, further increasing the time cost associated with these tools. This is in contrast to using PGS which would take only the amount of time to gather a sample of DNA and wait for results to come back. Although no comprehensive reports have been conducted on how much schools typically spend towards progress monitoring tools, some research has reported the costs of many popular assessments. Estimates show costs of progress monitoring tools to widely ranging from $6.00 per student per year to up to $55.00 per student per year^43^. Some progress monitoring tools offer other options, such as per teacher rates that can be as low as $30 per teacher per year, or per school rates^42^. These fees only include the use of the actual product, however. There are often several additional associated costs such as annual licensing fees as high as $500 or initial technology fees as high as $1700, data housing fees over $500 per school or an additional $4 per student, and training and upkeep fees reaching as high as $6600 per 30 people^42^. The cost for PGS is also somewhat unclear. Though commercial offerings for genetic sequencing are now as low as $100, the actual cost of genetic screening that would be of interest to educators is less known. Several options are available depending on the number of SNP’s, and the specific SNP’s every screener is focusing on, with widely ranging costs. There is also the question of how to analyze and house this data, which would incur additional costs to schools. One known benefit however for the cost of genetically screening students is that results could be reused for years to come, and in turn, the overall cost dispersed throughout a student’s entire school career. Before implementation of any PGS in school settings, comprehensive cost-benefit analyses should be conducted analyzing everything from current costs of progress monitoring tools to the potential overhead costs of implementing PGS and the benefit they would bring. After doing so, it may well be the case that schools may not value the additional incremental value of PGS as much as it costs to implement.

Another important factor to consider is the amount of shared variance predicted by PGS and progress monitoring (Supplementary Tables 1, 5, 9, and 13). Although this paper focused primarily on the incremental value of PGS in addition to existing progress monitoring tools, there is a significant amount of variance in end-of-year achievement as well as overlap in screener accuracy between PGS and progress monitoring. As such, as PGS gets better it will predict more and more of the variance also predicted by progress monitoring. Though it is unlikely that PGS would ever completely replace progress monitoring, it could alter the way in which current progress monitoring takes place. For example, it may be the case that rather than several waves of progress monitoring being necessary, the addition of PGS makes it so fewer would be sufficient.

There are significant concerns to consider with potential PGS implementation in schools. First, there is an issue of representation in the existing work that has the potential to constrain the current study findings. The reference samples used to calculate current PGS have largely been drawn from populations with European ancestry (67%) and East Asian ancestry (19%), with very few studies have used cohorts of individuals with African, Hispanic, or Indigenous ancestries (3.8%)^26,30^. Research has shown the commonly used PGS derived from these European ancestry populations have significantly less predictive power for individuals with non-European ancestry^26,30^. Therefore, before any conversation regarding the implementation of PGS in school settings is to take place, significantly more research needs to be conducted that includes individuals from non-European ancestry populations. Tied to this, a limitation of our work is that our results are only applicable for school children with European ancestry, as our estimates relied on the published effectiveness estimates drawn from those limited populations. The existing PGS has been built around samples containing a majority of individuals with European ancestral backgrounds, and research has shown PGS become less effective when applied to groups of different ancestral backgrounds than that from which they were developed^26,30^. It is likely the predictive power of PGS versus progress monitoring tools would be greatly decreased for individuals with other ancestries.

There are also grave ethical concerns tied with using genetic information in school settings mentioned prior. There is potential that genetic information will be used against children from marginalized groups. Particularly, the idea of scientific racism presents a large ethical concern and will require necessary conversations before any discussion of the implementation of PGS in schools^44^. Another ethical concern is in the way that using PGS is framed. PGS in its current state, just as with all existing screening systems for academic risk, points towards risk factors and probabilities, rather than an absolute diagnosis of any status or condition. If schools were to utilize PGS, they must accurately express to teachers and parents that the results are not absolute or diagnostic of learning disabilities. Interpreting the results as such could lead to potentially erroneous outcomes, with teachers and parents potentially viewing a student’s ability as pre-determined by their PGS. Research in the field of genomics has found that individuals tend to overemphasize the importance of genetics, leading to a deterministic and often fearful view of the results^3^. To combat this, genetic counseling has become common alongside genetic testing in the medical field^45^ and has been found to be effective in reducing anxiety and distress related to genetic test results as well as increasing accurate understanding of what genetic risk means^46,47^. For school settings, a similar approach to genetic counseling should be considered, as well as proper educational outreach and professional development with not only the members of the school but with all educational stakeholders connected to the school, including parents and the larger community. Further, issues related to genetic essentialism and biodeterminism can be tackled from an early age through instruction aimed at increasing genetic literacy^48,49^. This is likely only true however if said instruction is intelligently designed with a humane approach combatting these views in mind. If not, genetic instruction with only simple explanations of sex or population differences can actually increase belief in these ideas^50,51^.

Overall, it does appear as though PGS could have practical value in education settings, but that this value depends largely on several other factors, and must be weighed against ethical concerns. We found that though genetic screening may not be useful for tailoring education for individual students, it has the potential to provide utility as a time-sensitive and inexpensive additional screening tool.

## Methods

We sought to explore what benefit PGS could offer in a school setting by predicting student ability, above and beyond current progress monitoring efforts. To evaluate this potential impact, we took two approaches. For both approaches, we used assumptions of the multivariate normal distributions, as well as existing estimates of PGS and progress monitoring effectiveness. Assumptions of the multivariate normal distribution state that when multiple variables are normally distributed with one another, with no signs of heteroskedasticity, skewness, or other violations of normality, that knowledge of where an individual lies one variable is indicative of where that individual lies on the others. Research on several progress monitoring tests has found progress monitoring scores to be normally distributed with end-of-the-year achievement outcomes. A study by Hintze and Christ^52^ found that curriculum-based measurement (CBM) showed no signs of violating multivariate normality, even when varying assessment difficulty and grade level. Further, studies examining progress monitoring at the extremes of student achievement found progress monitoring to be equally as effective for gifted^53^, and low achieving students^54^. For PGS, it is assumed that a large number of genetic variants contribute to complex traits and that at the population level the distribution of these genetic variants and overall PGS are normally distributed^55,56^. The assumptions were tested by Krapohl et al.^57^, finding these assumptions to be accurate. The extent to which one accurately predicts another is the direct result of the correlation between the variables. When the correlation is strong, there is a more limited range of values that the second variable can take on given the individual’s value for the first variable, and in turn, the prediction is more accurate. When the correlation weakens, however, the range of possible values for the second variable greatly increases, and the prediction accuracy decreases. Thus, assuming multivariate normality, knowing the correlations among multiple variables can be used to directly estimate the overall and unique predictive powers of the variables on one another. These assumptions were carried into this paper and applied using estimates of the correlations between the variables of interest in attempts to answer the research questions.

The first research question aimed to understand what value PGS could add to predicting scores for an entire distribution of students. For this, an approach was taken that examined PGS’s effectiveness to predict the overall end of the year academic achievement and address the question of PGS could be used to increase educators’ ability to predict general education scores for all students. The second research question focused on if PGS could be used effectively as a screener tool for learning disabilities. For this, an approach was taken that used statistical software to mathematically compute cumulative distribution functions using various cut-points for learning disability classification. That is, mathematically determined how many individuals would get accurately screened into or out of learning disability services based on the effectiveness of PGS and the relations between PGS, progress monitoring scores, and end of the year achievement scores.

### Ethics note

Being that this study involved no human participants or their data of any sort and rather used simulated data, no formal ethics approval was required by a university or institutional review board for the completion of this study.

### Polygenic risk score effectiveness

The ability of PGS to predict educational outcomes has been studied and reported in recent literature. Current estimates, drawn from very large cumulative samples, of PGS’s ability to predict reading comprehension in early grades, range between 2% and 5%^25^. This 2% value was used to estimate the lowest range of effectiveness for PGS in this study, in particular allowing us to generalize to early subject-specific measures and for at-risk and minority populations for whom PGS are less effective. For general intelligence and educational attainment in later grades, these estimates are higher, with current ability to predict at around 10–15%, with new SNP chips and technologies being estimated to increase this up to 20% of the variation in intelligence in just the near future^13,27^. Here, 10% and 20% values represent the range of PGS’s ability to predict the more general outcomes (i.e., general cognitive ability and educational attainment vs reading performance), as well as provide a realistic ceiling for PGS effectiveness with current technology. As such, these estimates were also used in the subsequent analysis. Finally, behavioral genetics research has pointed to an overall heritability of around 50% for educational outcomes^13^. Although technologies are not yet able to predict educational outcomes at this level^58^ this represents a theoretical upper limit of PGS effectiveness^13^ and was the final value tested.

Square roots were taken of these 2%, 10%, 20%, and 50% values of variance predicted, to compute correlations between PGS and end of the year achievement. Since progress monitoring is also a measure of academic ability, the same correlations were used between PGS and progress monitoring as is used for PGS and end-of-year achievement. That is, for instances in which PGS predicts the end of the year reading achievement with 2% accuracy, it was also estimated that PGS would predict the reading achievement progress monitoring tests attempting to measure the same construct with a 2% accuracy as well. It could be the case that this is not necessarily the case, however, no data or studies are available that make the argument otherwise. Further values were tested for the level of PGS effectiveness including every 10 percentage points increase up to 80% to account for the variability and debatable nature of the actual maximum threshold of heritability. If PGS were to ever predict more than 80% of achievement, they would no doubt be useful if ethical concerns were adequately addressed.

### Progress monitoring effectiveness

The accuracy of commonly used screeners for student achievement varies widely on their ability to predict the standardized end-of-the-year achievement assessments across early elementary grades, with ranges between 0.33 and 0.76 when examining reading achievement^55–59^. For the values of progress monitoring effectiveness for both approaches, this paper used the median value of this range (i.e., 0.55) and upper and lower values 0.15 from this value (i.e., 0.40 and 0.70) to capture the bulk of the estimates for much popular progress monitoring assessments. Although this range was set by the literature focused on reading achievement, these values served as a good rule of thumb to anchor the values needed for progress monitoring predictive effectiveness. Using this range allowed us to examine what differences may arise in PGS’s incremental value when progress monitoring effectiveness is high or low.

### Research question 1

To answer the question of what incremental value PGS could have in generally predicting student academic achievement overall, a combination of data simulation and regression procedures was used. For this, data were simulated using the R “MASS” statistical package^60^ that was multivariate normally distributed with the different combinations of existing relations across variables based on prior research. That is, every combination of PGS effectiveness (2%, 10%, 20%, and 50%) and progress monitoring effectiveness (*r* = 0.40, 0.55, 0.70) was computed, for a total of 12 combinations of PGS and progress monitoring effectiveness. Further values were tested for the level of PGS effectiveness including every 10 percentage points increase up to 80% to account for the variability and debatable nature of the actual maximum threshold of heritability. Using this data a stepwise regression approach was used, in that first end-of-year academic achievement was regressed on both progress monitoring scores and PGS, and then again regressed only on progress monitoring scores. By doing so, the overall amount of variance predicted in end-of-year achievement by progress monitoring scores and PGS, dependent on the given effectiveness of each, was estimated. In addition, by comparing regression models, the unique variance predicted by each was determined. This comparison was important for understanding the value in predicting student scores that PGS could add beyond what current progress monitoring tools and strategies are already doing.

### Research question 2

The second approach taken was to examine the incremental value of PGS as a screener for learning disabilities. The multivariate normal distribution can be used to determine the prevalence and accuracy of predicting learning disabilities when the correlations among all variables are known, and the variables are an outcome variable by which learning disabilities can be measured (academic achievement) and independent variables with meaningful relations to said outcome variable (screener assessments or PGS)^61^. The cumulative distribution functions of complex multivariate normal distributions with many dimensions can only be exactly computed by hand, however extensive research has been conducted on the most accurate way to estimate these distributions^60–66^, and has resulted in several statistical packages effective at computing these estimates within 10–6 of the exact value when less than 5 dimensions are present. We used the method developed by Miwa and colleagues^65^ within the pmvnorm function in the R “mvtnorm” statistical package^67^ to evaluate the multivariate cumulative distributions of PGS, progress monitoring, and end-of-the-year achievement score. This method used the same estimates for PGS and progress monitoring effectiveness for correlations among the variables, but rather than simulating data, mathematically calculated the predictive values of PGS and progress monitoring for identifying students with learning disabilities. Different cutoffs (5th, 10th, 15th, and 20th percentile of achievement) for classification of a learning disability were tested to examine if PGS incremental value differs depending on how learning disabilities are defined. Since this is testing effectiveness as a screener, the cutoff for students being labeled at-risk by either PGS or progress monitoring was set at 10 percentile points higher than the cutoff for having said disability (i.e., students in the 20th percentile or lower of PGS or progress monitoring scores were identified as at-risk when learning disability is defined as being in the tenth or below percentile for end of the year achievement). Having a cut-off higher than the diagnostic criteria allows the false-negative rate to be reduced^68^, which is useful when the costs for false negatives are greater than the costs for false positives such as school settings^69^. This is not necessarily the way that all screeners work, and it could well be the case that for some schools at-risk cut-offs are set at the same cut-off as a diagnosis for a learning disability. To account for the difference in screener cut-offs and usage, a shinyapp was developed (https://jshero.shinyapps.io/two_predictor_screener/) allowing for any combination of screener and diagnosis cut-offs to be tested for any combination of screening tools with knowable or able to be estimated correlations between said screeners and the outcome of interest. Further, to account for the variability and debatable nature of the actual maximum threshold of heritability additional values were tested for the level of PGS effectiveness including every 10 percentage points increase up to 80% effectiveness for one model (20th percentile cutoff).

For every combination of PGS and progress monitoring, the cumulative distribution provided 8 resulting values.

Result 1—a student’s end-of-the-year achievement score classifies them as having a learning disability, and both PGS and progress monitoring accurately predicted this. This is a shared true positive.

Result 2—a student’s end-of-the-year achievement score classifies them as having a learning disability, but only PGS accurately predicted this. This is a unique true positive for PGS and a false negative for progress monitoring.

Result 3—a student’s end-of-the-year achievement score classifies them as having a learning disability, but only progress monitoring accurately predicted this. This is a unique true positive for progress monitoring and a false negative for PGS.

Result 4—a student’s end-of-the-year achievement score classifies them as having a learning disability, but neither PGS nor progress monitoring accurately predicted this. This is a shared false negative.

Result 5—a student’s end-of-the-year achievement score classifies them as not having a learning disability, and both PGS and progress monitoring accurately predicted this. This is a shared true negative.

Result 6—a student’s end-of-the-year achievement score classifies them as not having a learning disability, but only PGS accurately predicted this. This is a unique true negative for PGS and a false positive for progress monitoring.

Result 7—a student’s end-of-the-year achievement score classifies them as not having a learning disability, but only progress monitoring accurately predicted this. This is a unique true negative for progress monitoring and a false positive for PGS.

Result 8—a student’s end-of-the-year achievement score classifies them as not having a learning disability, but neither PGS nor progress monitoring accurately predicted this. This is a shared false positive.

These resulting values were then computed into PPVs and NPVs. That is, PPV representing the percentage of students identified as at-risk that ended up classified as having a learning disability, and NPV representing the percentage of students identified as not at-risk that did not end up classified as having a learning disability. PPV and NPV were computed separately for the three different following scenarios: (1) students meeting the progress monitoring cutoff were classified as at-risk, (2) students meeting the cutoff for either PGS or progress monitoring were classified as at-risk, and (3) students meeting the cutoff for both PGS and progress monitoring were classified as at-risk. These distinctions were made to provide a baseline of current predictive values (scenario 1), and to reflect that some schools may value a broader screener (scenario 2), whereas others want to make classification more strict (scenario 3).

### Model assumptions

Across all analyses and for each of the two sets of analyses corresponding to each research question, model assumptions were made. Across both sets of analyses, assumptions of multivariate normality were made. That is, if we collected data on the constructs modeled in this paper, they would have a univariate distribution showing no signs of skewness or kurtosis, and a multivariate distribution showing no signs of heteroskedasticity. Based on the research included in the prior sections^52–57^ we felt justified in this assumption. Second, both sets of models assume that progress monitoring tools and PGS would work additively to predict the end of the year achievement scores. That is, progress monitoring and PGS will both individually predict the end of the year student achievement, with a certain proportion of their individual levels of prediction being unique and the other proportion being shared. The unique variance predicted by each was a focal point in the results for the first research question but was not heavily discussed for the second. The reason for this is that it is unlikely that PGS be used on its own to screen students. As such, understanding what PGS adds to current screening efforts could be discussed without needing a discussion of unique variance predicted. The unique variance predicted by PGS related to the prediction of learning disabilities was calculated and is presented in the supplemental tables.

Another key assumption of the modeling is that PGS is equally correlated with end-of-the-year student achievement as it is with progress monitoring. There is potential that these values are different, however, there simply is no research to support this or suggest what values would be appropriate to use otherwise. To address this, a sensitivity analysis was conducted for one combination of PGS and progress monitoring values to see how results would change if this assumption were to be violated. For this sensitivity analysis we chose the 20th percentile as the cutoff due to this cutoff having the largest range of PPV and NPV’s in the original analyses, and in turn, had the largest potential impact when violating this assumption. The results from the sensitivity analyses suggested that changes were negligible, with PPV only being impacted for the “either cutoff” result, and NPV only being impacted for the ‘both cutoffs’ result. When PGS was less correlated with progress monitoring as compared to end-of-year achievement, this impacted the PPV when meeting either cutoff by <5%, and impacted NPV when meeting both cutoffs by <3% when PGS was almost entirely uncorrelated with progress monitoring scores. This represents the best-case scenario for proponents of using PGS in school settings, as less variance predicted at the end of year achievement by PGS and progress monitoring will be shared. When PGS were more correlated with progress monitoring scores than end-of-the-year achievement, results again were only significantly shifted for the PPV “either cutoffs” and NPV “both cutoffs” results. Here, if PGS and progress monitoring were correlated at 0.9, this led to changes of ~13% and ~7% for PPV and NPV respectively. This is the worst-case scenario for advocates of using PGS in classrooms, with a higher correlation between progress monitoring and end-of-year achievement meaning the PGS would be less effective.

A final assumption was that the correlation between PGS and end-of-year achievement would hold constant after the initial screening and intervention took place. It is certainly possible that once students were identified as being genetically at-risk for a learning disability and interventions were imposed, that the relation between PGS and end-of-the-year achievement would deteriorate. This is because the PGS value would remain constant, but scores, in theory, would increase due to the intervention. The analyses presented within this paper do not address this potential dynamic situation. This idea however was one driving factor for focusing on both PGS as an additional screener for learning disabilities, and early intervention and use of PGS. Even if PGS became less correlated with end-of-the-year achievement for those students who had received the intervention, PGS would have indeed proven to be useful when used as an early screener for learning disability risk, as children would have grown in achievement scores, the outcome of interest. Further, using PGS is a tool part of personalized education early in education, done at the most beneficial time for intervention^70,71^, and before any of these dynamic influences, is a benefit of using PGS.

### Reporting summary

Further information on research design is available in the Nature Research Reporting Summary linked to this article.

## Supplementary information

Supplementary Information

Reporting Summary

## Data Availability

This project utilized simulated data. Code to reproduce said simulated data is available at (https://github.com/jshero/Polygenic-Score-Utility). Other correspondence can be directed to J.A.S. at shero@psy.fsu.edu

## Code availability

All code for simulating and analyzing data available at (https://github.com/jshero/Polygenic-Score-Utility). Accompanying ShinyApp for further analysis available at (https://jshero.shinyapps.io/two_predictor_screener/). Other correspondence can be directed to J.A.S. at shero@psy.fsu.edu
