# Supplementary material for: The practical utility of genetic screening in school settings
Source: NPJ Sci Learn. 2021 Jun 1;6:12. doi: 10.1038/s41539-021-00090-y (PMC8169884; doi:10.1038/s41539-021-00090-y)
Supplement: Supplementary file 1 — Supplementary Information [file 41539_2021_90_MOESM1_ESM.pdf]

This file includes:

Tables: Supplementary Table 1 to Supplementary Table 19

5th percentile cutoff												
	Progress Monitoring r=0.40				Progress Monitoring r=0.55				Progress Monitoring r=0.70			
**PGS Explained Variance**	2%	10%	20%	50%	2%	10%	20%	50%	2%	10%	20%	50%
Shared TP	0.01	0.01	0.01	0.02	0.01	0.01	0.01	0.02	0.01	0.01	0.02	0.03
PGS only TP	0.01	0.01	0.01	0.02	0.00	0.01	0.01	0.01	0.00	0.00	0.00	0.01
Progress Monitoring Only TP	0.02	0.01	0.01	0.00	0.02	0.02	0.01	0.00	0.03	0.02	0.02	0.01
Shared FN	0.02	0.02	0.02	0.01	0.02	0.02	0.01	0.01	0.01	0.01	0.01	0.01
Shared FP	0.03	0.03	0.04	0.06	0.02	0.03	0.04	0.06	0.02	0.03	0.04	0.05
Progress monitoring only TN	0.11	0.10	0.09	0.05	0.11	0.10	0.09	0.06	0.12	0.10	0.09	0.06
PGS only TN	0.10	0.10	0.09	0.07	0.10	0.09	0.08	0.07	0.09	0.09	0.08	0.06
Shared TN	0.71	0.72	0.73	0.77	0.71	0.73	0.74	0.77	0.72	0.73	0.74	0.77

Supplementary Table 1. Distributed probabilities of PGS and progress monitoring tools used to screen for learning disability risk (5th percentile cutoff). Proportion of distributions shown that meet each category, columns add up to approximately 1.

Predictive Power of only Progress Monitoring (5th percentile cutoff)		
Progress Monitoring		
**0.40**	**0.55**	**0.70**
14% (97%)	18% (98%)	23% (98%)

Supplementary Table 2. Positive and negative predictive value of progress monitoring tools as a screener for learning disabilities (5th percentile or lower in achievement*)*. Positive predictive value presented as a percentage with negative predictive values presented as a percentage in parentheses below.

		Predictive Power when Meeting either Cutoff (5th percentile cutoff)		
		Progress Monitoring		
		**0.40**	**0.55**	**0.70**
PGS Predictive Power	**2%**	10% (97%)	12% (98%)	14% (98%)
	**10%**	11% (97%)	13% (98%)	15% (99%)
	**20%**	13% (97%)	14% (98%)	16% (99%)
	**50%**	17% (98%)	18% (99%)	19% (99%)

Supplementary Table 3. Positive and negative predictive value of progress monitoring tools and PGS (meeting either's criteria) as a screener for learning disabilities (5th percentile or lower in achievement). Positive predictive value presented as a percentage with negative predictive values presented as a percentage in parentheses below.

		Predictive Power when Meeting Both Cutoffs (5th percentile cutoff)		
		Progress Monitoring		
		**0.40**	**0.55**	**0.70**
PGS Predictive Power	**2%**	10% (94%)	22% (94%)	27% (95%)
	**10%**	20% (95%)	25% (95%)	31% (95%)
	**20%**	22% (95%)	28% (95%)	33% (96%)
	**50%**	24% (96%)	30% (97%)	35% (97%)

Supplementary Table 4. Positive and negative predictive value of progress monitoring tools and PGS (meeting criteria of both) as a screener for learning disabilities (5th percentile or lower in achievement). Positive predictive value presented as a percentage with negative predictive values presented as a percentage in parentheses below.

10th percentile cutoff												
	Progress Monitoring r=0.40				Progress Monitoring r=0.55				Progress Monitoring r=0.70			
**PGS Explained Variance**	2%	10%	20%	50%	2%	10%	20%	50%	2%	10%	20%	50%
Shared TP	0.01	0.02	0.03	0.04	0.02	0.02	0.03	0.05	0.02	0.03	0.04	0.05
PGS only TP	0.01	0.02	0.02	0.03	0.01	0.01	0.02	0.02	0.01	0.01	0.01	0.02
Progress Monitoring Only TP	0.03	0.02	0.02	0.00	0.04	0.03	0.02	0.01	0.05	0.04	0.03	0.01
Shared FN	0.04	0.04	0.04	0.03	0.03	0.03	0.03	0.02	0.02	0.02	0.02	0.02
Shared FP	0.04	0.05	0.05	0.07	0.04	0.04	0.05	0.07	0.03	0.04	0.04	0.06
Progress monitoring only TN	0.13	0.11	0.10	0.06	0.14	0.12	0.10	0.06	0.14	0.12	0.11	0.07
PGS only TN	0.12	0.11	0.10	0.08	0.11	0.10	0.09	0.08	0.10	0.09	0.09	0.07
Shared TN	0.61	0.63	0.65	0.69	0.62	0.64	0.65	0.69	0.63	0.65	0.66	0.70

Supplementary Table 5. Distributed probabilities of PGS and progress monitoring tools used to screen for learning disability risk (10th percentile cutoff). Proportion of distributions shown that meet each category, columns add up to approximately 1.

Predictive Power of only Progress Monitoring (10th percentile cutoff)		
Progress Monitoring		
**0.40**	**0.55**	**0.70**
22% (93%)	28% (94%)	34% (96%)

Supplementary Table 6. Positive and negative predictive value of progress monitoring tools as a screener for learning disabilities (10th percentile or lower in achievement*).* Positive predictive value presented as a percentage with negative predictive values presented as a percentage in parentheses below.

		Predictive Power when Meeting either Cutoff (10th percentile cutoff)		
		Progress Monitoring		
		**0.40**	**0.55**	**0.70**
PGS Predictive Power	**2%**	17% (94%)	19% (95%)	22% (96%)
	**10%**	19% (94%)	21% (95%)	24% (97%)
	**20%**	20% (95%)	23% (96%)	25% (97%)
	**50%**	26% (96%)	27% (97%)	29% (98%)

Supplementary Table 7. Positive and negative predictive value of progress monitoring tools and PGS (meeting either's criteria) as a screener for learning disabilities (10th percentile or lower in achievement). Positive predictive value presented as a percentage with negative predictive values presented as a percentage in parentheses below.

		Predictive Power when Meeting Both Cutoffs (10th percentile cutoff)		
		Progress Monitoring		
		**0.40**	**0.55**	**0.70**
PGS Predictive Power	**2%**	26% (88%)	32% (88%)	38% (89%)
	**10%**	30% (89%)	36% (89%)	42% (90%)
	**20%**	33% (90%)	38% (91%)	45% (91%)
	**50%**	35% (92%)	41% (93%)	48% (94%)

Supplementary Table 8. Positive and negative predictive value of progress monitoring tools and PGS (meeting criteria of both) as a screener for learning disabilities (10th percentile or lower in achievement). Positive predictive value presented as a percentage with negative predictive values presented as a percentage in parentheses below.

15th percentile cutoff												
	Progress Monitoring r=0.40				Progress Monitoring r=0.55				Progress Monitoring r=0.70			
**PGS Explained Variance**	2%	10%	20%	50%	2%	10%	20%	50%	2%	10%	20%	50%
Shared TP	0.03	0.04	0.04	0.06	0.03	0.04	0.05	0.07	0.04	0.05	0.06	0.08
PGS only TP	0.02	0.03	0.03	0.04	0.02	0.02	0.02	0.03	0.01	0.02	0.02	0.02
Progress Monitoring Only TP	0.05	0.04	0.03	0.01	0.06	0.05	0.04	0.01	0.07	0.06	0.05	0.02
Shared FN	0.06	0.05	0.05	0.04	0.04	0.04	0.04	0.03	0.03	0.03	0.03	0.02
Shared FP	0.05	0.06	0.07	0.09	0.05	0.06	0.06	0.08	0.04	0.05	0.05	0.07
Progress monitoring only TN	0.15	0.13	0.11	0.06	0.15	0.13	0.11	0.07	0.16	0.14	0.12	0.08
PGS only TN	0.13	0.12	0.11	0.09	0.12	0.11	0.10	0.09	0.10	0.10	0.09	0.08
Shared TN	0.52	0.55	0.57	0.61	0.53	0.56	0.57	0.62	0.55	0.57	0.59	0.63

Supplementary Table 9. Distributed probabilities of PGS and progress monitoring tools used to screen for learning disability risk (15th percentile cutoff). Proportion of distributions shown that meet each category, columns add up to approximately 1.

Predictive Power of only Progress Monitoring (15th percentile cutoff)		
Progress Monitoring		
**0.40**	**0.55**	**0.70**
29% (90%)	35% (92%)	42% (94%)

Supplementary Table 10. Positive and negative predictive value of progress monitoring tools as a screener for learning disabilities (15th percentile or lower in achievement*)*.
Positive predictive value presented as a percentage with negative predictive values presented as a percentage in parentheses below.

		Predictive Power when Meeting either Cutoff (15th percentile cutoff)		
		Progress Monitoring		
		**0.40**	**0.55**	**0.70**
PGS Predictive Power	**2%**	22% (90%)	25% (92%)	28% (94%)
	**10%**	25% (91%)	27% (93%)	30% (95%)
	**20%**	27% (92%)	29% (94%)	32% (95%)
	**50%**	32% (94%)	34% (95%)	36% (96%)

Supplementary Table 11. Positive and negative predictive value of progress monitoring tools and PGS (meeting either's criteria) as a screener for learning disabilities (15th percentile or lower in achievement). Positive predictive value presented as a percentage with negative predictive values presented as a percentage in parentheses below.

		Predictive Power when Meeting Both Cutoffs (15th percentile cutoff)		
		Progress Monitoring		
		**0.40**	**0.55**	**0.70**
PGS Predictive Power	**2%**	33% (81%)	39% (82%)	46% (83%)
	**10%**	37% (83%)	43% (84%)	50% (85%)
	**20%**	40% (84%)	46% (85%)	52% (87%)
	**50%**	43% (88%)	49% (89%)	55% (90%)

Supplementary Table 12. Positive and negative predictive value of progress monitoring tools and PGS (meeting criteria of both) as a screener for learning disabilities (15th percentile or lower in achievement). Positive predictive value presented as a percentage with negative predictive values presented as a percentage in parentheses below.

20th percentile cutoff												
	Progress Monitoring r=0.40				Progress Monitoring r=0.55				Progress Monitoring r=0.70			
**PGS Explained Variance**	2%	10%	20%	50%	2%	10%	20%	50%	2%	10%	20%	50%
Shared TP	0.04	0.06	0.07	0.09	0.05	0.06	0.08	0.10	0.06	0.07	0.09	0.12
PGS only TP	0.03	0.04	0.04	0.05	0.03	0.03	0.03	0.04	0.02	0.02	0.02	0.03
Progress Monitoring Only TP	0.06	0.05	0.04	0.01	0.07	0.06	0.05	0.02	0.09	0.07	0.06	0.03
Shared FN	0.06	0.06	0.06	0.05	0.05	0.05	0.05	0.04	0.04	0.04	0.03	0.03
Shared FP	0.07	0.07	0.08	0.10	0.06	0.07	0.07	0.09	0.05	0.06	0.06	0.08
Progress monitoring only TN	0.16	0.13	0.11	0.06	0.17	0.14	0.12	0.07	0.17	0.15	0.13	0.08
PGS only TN	0.13	0.12	0.12	0.10	0.12	0.11	0.11	0.09	0.10	0.10	0.09	0.08
Shared TN	0.44	0.47	0.49	0.55	0.46	0.48	0.50	0.55	0.47	0.50	0.52	0.56

Supplementary Table 13. Distributed probabilities of PGS and progress monitoring tools used to screen for learning disability risk (20th percentile cutoff). Proportion of distributions shown that meet each category, columns add up to approximately 1.

Predictive Power of only Progress Monitoring (20th percentile cutoff)		
Progress Monitoring		
**0.40**	**0.55**	**0.70**
34% (86%)	41% (89%)	48% (92%)

Supplementary Table 14. Positive and negative predictive value of progress monitoring tools as a screener for learning disabilities (20th percentile or lower in achievement*).* Positive predictive value presented as a percentage with negative predictive values presented as a percentage in parentheses below.

		Predictive Power when Meeting either Cutoff (20th percentile cutoff)		
		Progress Monitoring		
		**0.40**	**0.55**	**0.70**
PGS Predictive Power	**2%**	28% (87%)	30% (90%)	33% (93%)
	**10%**	30% (89%)	32% (91%)	35% (93%)
	**20%**	32% (90%)	34% (92%)	37% (94%)
	**50%**	38% (92%)	40% (94%)	42% (95%)

Supplementary Table 15. Positive and negative predictive value of progress monitoring tools and PGS (meeting either's criteria) as a screener for learning disabilities (20th percentile or lower in achievement). Positive predictive value presented as a percentage with negative predictive values presented as a percentage in parentheses below.

		Predictive Power when Meeting Both Cutoffs (20th percentile cutoff)		
		Progress Monitoring		
		**0.40**	**0.55**	**0.70**
PGS Predictive Power	**2%**	38% (74%)	45% (75%)	51% (76%)
	**10%**	43% (77%)	49% (78%)	55% (79%)
	**20%**	45% (79%)	51% (80%)	57% (82%)
	**50%**	49% (84%)	54% (85%)	61% (87%)

Supplementary Table 16. Positive and negative predictive value of progress monitoring tools and PGS (meeting criteria of both) as a screener for learning disabilities (20th percentile or lower in achievement). Positive predictive value presented as a percentage with negative predictive values presented as a percentage in parentheses below.

		Variance in End of Year Achievement Predicted by Progress Monitoring and PGS		
		Progress Monitoring Effectiveness		
		**0.40**	**0.55**	**0.70**
PGS Predictive Power	**2%**	0.167 (0.007)	0.306 (0.004)	0.492 (0.002)
	**10%**	0.199 (0.040)	0.325 (0.023)	0.500 (0.010)
	**20%**	0.249 (0.089)	0.353 (0.051)	0.513 (0.023)
	**30%**	0.315 (0.155)	0.389 (0.086)	0.529 (0.039)
	**40%**	0.399 (0.239)	0.437 (0.134)	0.550 (0.060)
	**50%**	0.5198 (0.359)	0.505 (0.203)	0.580 (0.090)
	**60%**	0.701 (0.541)	0.607 (0.304)	0.625 (0.135)
	**70%**	0.700 (NPD)	0.776 (0.473)	0.700 (0.210)
	**80%**	0.800 (NPD)	0.997 (0.695)	0.799 (0.309)

Supplementary Table 17. Variance in End of Year Achievement Predicted by Progress Monitoring and PGS (Extended). R^2 values presented, with unique contribution of R^2 by PGS presented in parentheses. NPD- Not positive definite, unique variance predicted by PGS would exceed overall variance predicted.

		Predictive Power when Meeting either Cutoff (20th percentile cutoff)		
		Progress Monitoring		
		**0.40**	**0.55**	**0.70**
PGS Predictive Power	**2%**	28% (87%)	30% (90%)	33% (93%)
	**10%**	30% (89%)	32% (91%)	35% (93%)
	**20%**	32% (90%)	34% (92%)	37% (94%)
	**30%**	34% (91%)	36% (92%)	39% (94%)
	**40%**	36% (91%)	38% (93%)	40% (95%)
	**50%**	38% (92%)	40% (94%)	42% (95%)
	**60%**	40% (93%)	42% (94%)	44% (96%)
	**70%**	NA	45% (95%)	46% (96%)
	**80%**	NA	NA	49% (97%)

Supplementary Table 18. Positive and negative predictive value of progress monitoring tools and PGS (meeting either's criteria) as a screener for learning disabilities (20th percentile or lower in achievement) (Extended). Positive predictive value presented as a percentage with negative predictive values presented as a percentage in parentheses below. NA- Not mathematically possible within model constraints.

		Predictive Power when Meeting either Cutoff (20th percentile cutoff)		
		Progress Monitoring		
		**0.40**	**0.55**	**0.70**
PGS Predictive Power	**2%**	38% (74%)	45% (75%)	51% (76%)
	**10%**	43% (77%)	49% (78%)	55% (79%)
	**20%**	45% (79%)	51% (80%)	57% (82%)
	**30%**	47% (81%)	53% (82%)	59% (84%)
	**40%**	48% (82%)	54% (84%)	60% (85%)
	**50%**	49% (84%)	54% (85%)	61% (87%)
	**60%**	49% (85%)	55% (87%)	61% (89%)
	**70%**	NA	54% (88%)	61% (90%)
	**80%**	NA	NA	60% (91%)

Supplementary Table 19. Positive and negative predictive value of progress monitoring tools and PGS (meeting criteria of both) as a screener for learning disabilities (20th percentile or lower in achievement) (Extended). Positive predictive value presented as a percentage with negative predictive values presented as a percentage in parentheses below. NA- Nott mathematically possible within model constraints.
