# Supplementary material for: The practical utility of genetic screening in school settings
Source: NPJ Sci Learn. 2021 Jun 1;6:12. doi: 10.1038/s41539-021-00090-y (PMC8169884; doi:10.1038/s41539-021-00090-y)
Supplement: Supplementary file 2 — Reporting Summary [file 41539_2021_90_MOESM2_ESM.pdf]

nature research

Corresponding author(s): DBPR NPJSCILEARN-00388

Last updated by author(s): Dec 18, 2020

Reporting Summary

Nature Research wishes to improve the reproducibility of the work that we publish. This form provides structure for consistency and transparency in reporting. For further information on Nature Research policies, see our Editorial Policies and the Editorial Policy Checklist.

Statistics

For all statistical analyses, confirm that the following items are present in the figure legend, table legend, main text, or Methods section.

n/a	Confirmed	
☒	☐	The exact sample size (n) for each experimental group/condition, given as a discrete number and unit of measurement
☒	☐	A statement on whether measurements were taken from distinct samples or whether the same sample was measured repeatedly
☐	☒	The statistical test(s) used AND whether they are one- or two-sided<br>*Only common tests should be described solely by name; describe more complex techniques in the Methods section.*
☒	☐	A description of all covariates tested
☐	☒	A description of any assumptions or corrections, such as tests of normality and adjustment for multiple comparisons
☒	☐	A full description of the statistical parameters including central tendency (e.g. means) or other basic estimates (e.g. regression coefficient) AND variation (e.g. standard deviation) or associated estimates of uncertainty (e.g. confidence intervals)
☒	☐	For null hypothesis testing, the test statistic (e.g. F, t, r) with confidence intervals, effect sizes, degrees of freedom and P value noted<br>*Give P values as exact values whenever suitable.*
☒	☐	For Bayesian analysis, information on the choice of priors and Markov chain Monte Carlo settings
☒	☐	For hierarchical and complex designs, identification of the appropriate level for tests and full reporting of outcomes
☐	☒	Estimates of effect sizes (e.g. Cohen's d, Pearson's r), indicating how they were calculated

Our web collection on statistics for biologists contains articles on many of the points above.

Software and code

Policy information about availability of computer code

Data collection	This data was conducted using simulated data, and as such no traditional "data collection" occurred. Rather, data was simulated using the most recent version of R-Studio at the time of project completion. Specifically, the "MASS" and "mvtnorm" packages within R-Studio werre used for all simulations. All code for simulating and analyzing data available at (https://github.com/jshero/Polygenic-Score-Utility).
Data analysis	Data was analyzed using the most recent version of R-Studio at the time of project completion. Specifically, the "MASS" and "mvtnorm" packages within R-Studio were used for all analyses. All code for simulating and analyzing data available at (https://github.com/jshero/Polygenic-Score-Utility).

For manuscripts utilizing custom algorithms or software that are central to the research but not yet described in published literature, software must be made available to editors and reviewers. We strongly encourage code deposition in a community repository (e.g. GitHub). See the Nature Research guidelines for submitting code & software for further information.

Data

Policy information about availability of data

All manuscripts must include a data availability statement. This statement should provide the following information, where applicable:

- Accession codes, unique identifiers, or web links for publicly available datasets
- A list of figures that have associated raw data
- A description of any restrictions on data availability

All code for simulating and analyzing data available at (https://github.com/jshero/Polygenic-Score-Utility).

Field-specific reporting

Please select the one below that is the best fit for your research. If you are not sure, read the appropriate sections before making your selection.

☒ Life sciences ☐ Behavioural & social sciences ☐ Ecological, evolutionary & environmental sciences

For a reference copy of the document with all sections, see nature.com/documents/nr-reporting-summary-flat.pdf

Life sciences study design

All studies must disclose on these points even when the disclosure is negative.

Sample size	No "real data" was used, rather data was simulated. As a result, we were able to simulate several different datasets that had the statistical and relational qualities we were looking for, each with 100,000 data-points.
Data exclusions	Data was simulated, and as such there was no need to exclude any of the data-points. Data was simulated according to a predetermined structure, and as such all data-points were relevant to the research at hand.
Replication	Data was simulated and as such replicating the steps taken resulted in identical results. Code for the data simulation and analyses are available to other researchers, as well as an R ShinyApp, to allow other researchers to replicate and expand upon the results of the study.
Randomization	This section is not relevant to the study as data was simulated, and therefor there was no need or way for randomization to occur.
Blinding	Again, data was simulated so there was no need for blinding to occur.

Reporting for specific materials, systems and methods

We require information from authors about some types of materials, experimental systems and methods used in many studies. Here, indicate whether each material, system or method listed is relevant to your study. If you are not sure if a list item applies to your research, read the appropriate section before selecting a response.

Materials & experimental systems

n/a	Involved in the study	
☒	☐	Antibodies
☒	☐	Eukaryotic cell lines
☒	☐	Palaeontology and archaeology
☒	☐	Animals and other organisms
☒	☐	Human research participants
☒	☐	Clinical data
☒	☐	Dual use research of concern

Methods

n/a	Involved in the study	
☒	☐	ChIP-seq
☒	☐	Flow cytometry
☒	☐	MRI-based neuroimaging
